# Cholesterol regulation of PIP_2_: why cell type is so important

**DOI:** 10.3389/fphys.2012.00492

**Published:** 2013-01-07

**Authors:** Domenico M. Taglieri, Dawn A. Delfín, Michelle M. Monasky

**Affiliations:** ^1^Department of Pharmacology, College of Medicine, University of Illinois at ChicagoChicago, IL, USA; ^2^Department of Molecular and Cellular Biochemistry, College of Medicine, The Ohio State UniversityColumbus, OH, USA; ^3^Department of Physiology and Biophysics and Center for Cardiovascular Research, College of Medicine, University of Illinois at ChicagoChicago, IL, USA

**A commentary on**

**How cholesterol regulates endothelial biomechanics**

by Hong, Z., Staiculescu, M. C., Hampel, P., Levitan, I., and Forgacs, G. (2012). Front. Physio. 3:426. doi: 10.3389/fphys.2012.00426

Phosphatidylinositol 4,5-bisphosphate (PIP_2_) is a phospholipid found in cell membranes, and has been indicated to play important roles in cytoskeletal organization, cell motility, transduction of extracellular signals, regulation of ion channels at the plasma membrane, endocytosis, phagocytosis, and endosome function. It has also been linked to cancer in humans (Di Paolo and De Camilli, 2006). PIP_2_ can be hydrolyzed by membrane-bound phospholipase C beta into two second messengers, IP3 and diacylglycerol (DAG). Plasmalemmal cholesterol has been demonstrated to regulate PIP_2_ hydrolysis and thus its cellular function in skin fibroblasts and pancreatic β-cells (Kwik et al., 2003; Hao and Bogan, 2009).

In this original research article, Hong et al. (2012) address the role of plasmalemmal cholesterol in regulating the localization and metabolism of PIP_2_ in endothelial cells and the result on cell stiffness. They suggest that a decrease in cholesterol leads to disruption of PIP_2_ hydrolysis, which in turn results in increased cross-links between the membrane and cytoskeleton via PIP_2_ and increased cell stiffness. These results are important for understanding certain diseases, such as atherosclerosis, in which cholesterol levels are central to the pathology.

The mechanism by which cholesterol regulates PIP_2_ in the plasma membrane may not be the same for different cell types. Cholesterol depletion in fibroblasts leads to decreased levels of PIP_2_ in the plasma membrane, diminished membrane-cytoskeletal attachments, and decreased lateral motility (Kwik et al., 2003). Similarly, in cultured pancreatic β-cells, cholesterol depletion stimulates the hydrolysis of PIP_2_, thus reducing the amount of PIP_2_ at the plasma membrane (Hao and Bogan, 2009). These findings are in contrast to a study using HEK293 cells, in which membrane cholesterol enrichment promoted PIP_2_ depletion (Chun et al., 2010). Cholesterol depletion in lymphoblasts results in decreased lateral mobility of membrane proteins (Kwik et al., 2003). PIP_2_ lateral mobility has been described as low in atrial myocytes, and high in HEK293 cells and fibroblasts (Epand, 2008). Therefore, cholesterol may regulate PIP_2_ differently in various cell types.

Compartmentalization and regulation of PIP_2_ metabolism within the plasma membrane may contribute to differences observed between cell types (Epand, 2008; Kwiatkowska, 2010). Cholesterol-rich membrane microdomains (lipid rafts), which are known to be intimately involved in regulating a variety of G-protein coupled receptor-mediated functions, including those regulating PIP_2_ metabolism (Allen et al., 2007), have bidirectional relationship with actin cytoskeleton. In response to external stimuli, G_αq/11_ subunits stimulate membrane-bound phospholipase C beta, which then cleaves PIP_2_ into its two second messengers. Therefore, it is believed that the relationship among lipid raft-associated PIP_2_, G-proteins, and actin has strong implications in regulating actin assembly to modify cell shape and function. Conversely, actin associates with rafts and caveolae either as polymerized structures or as actin monomers, which might help to organize lipid raft domains and the molecules that are present in this structure to evoke a variety of cell signaling pathways in the cell interior (Caroni, 2001). Another exciting mechanism involves three proteins, namely GAP43, MARCKS, and CAP23, which accumulate at rafts, where they associate with PIP_2_, and promote its retention and clustering. By modulating PIP_2_ at plasmalemmal rafts, GAP43, MARCKS, and CAP23 regulate cell cortex actin dynamics through a common mechanism. It is believed that, in response to local signals, these proteins dissociate from PIP_2_, creating local pools of free PIP_2_, which result in diverse intracellular responses.

Nonetheless, the mechanisms through which PIP_2_ effector molecules mediate the various cellular responses to localized liberation of PIP_2_ are mostly unknown. Investigations in this direction will foster our understanding of cholesterol-mediated PIP_2_ intracellular functions.
